# Dual role of FGF in proliferation and endoreplication of *Drosophila* tracheal adult progenitor cells

**DOI:** 10.1093/jmcb/mjz055

**Published:** 2019-06-25

**Authors:** Cristina de Miguel, Josefa Cruz, David Martín, Xavier Franch-Marro

**Affiliations:** Institute of Evolutionary Biology (Consejo Superior de Investigaciones Científicas–Universitat Pompeu Fabra), Passeig Marítim de la Barceloneta 37, 08003 Barcelona, Spain

**Keywords:** FGF, trachea, adult progenitors, cut, proliferation, endoreplication, Pnt, Fzr

## Abstract

Adult progenitor cells activation is a key event in the formation of adult organs. In *Drosophila*, formation of abdominal adult trachea depends on the specific activation of tracheal adult progenitors (tracheoblasts) at the Tr4 and Tr5 spiracular branches. Proliferation of these tracheoblasts generates a pool of tracheal cells that migrate toward the posterior part of the trachea by the activation of the branchless/fibroblast growth factor (Bnl/FGF) signaling to form the abdominal adult trachea. Here, we show that, in addition to migration, Bnl/FGF signaling, mediated by the transcription factor Pointed, is also required for tracheoblast proliferation. This tracheoblast activation relies on the expression of the FGF ligand *bnl* in their nearby branches. Finally, we show that, in the absence of the transcription factor Cut (Ct), Bnl/FGF signaling induces endoreplication of tracheoblasts partially by promoting *fizzy-related* expression. Altogether, our results suggest a dual role of Bnl/FGF signaling in tracheoblasts, inducing both proliferation and endoreplication, depending on the presence or absence of the transcription factor Ct*,* respectively.

## Introduction

The formation of adult organs depends on the activation of progenitor undifferentiated cells during development. The temporal regulation of transcriptional activity of adult progenitor cells is critical to coordinate their proliferation and differentiation in order to form adult functional tissues. Although great progress has been achieved in the identification of signals that regulate the activity of progenitor cells, the characterization of the mechanisms underlying the temporal and spatial control of such events remains far from understood. Here, we use the formation of the adult tracheal system of *Drosophila*, the tubular organ responsible for oxygen transport (Klämbt et al., 1992; Samakovlis et al., 1996), to address this issue.

The embryonic trachea of *Drosophila* develops from 10 bilaterally symmetric clusters (Tr1–Tr10) of ~ 80 cells that invaginate to form epithelial sacs that remain connected to the epidermis through the spiracular branches (SBs) (Ghabrial et al., 2003). These cells migrate and differentiate under the control of the branchless/fibroblast growth factor (Bnl/FGF) signaling pathway during embryogenesis to generate a network of interconnected tubes that will function as the larval tracheal system. This larval tracheal network is then remodeled during pupal metamorphosis from a reduced number of different adult precursors cells, called tracheoblasts (Whitten, 1957; Manning and Krasnow, 1993; Samakovlis et al., 1996; Sato and Kornberg, 2002; Cabernard and Affolter, 2005; Guha and Kornberg, 2005; Guha et al., 2008; Sato et al., 2008; Weaver and Krasnow, 2008; Pitsouli and Perrimon, 2010; Djabrayan et al., 2014; Cruz et al., 2015). One particular type is the abdominal SB tracheoblasts, which are multipotent undifferentiated cells that are specified in the embryo and remain quiescent until the third larval instar (L3), when they proliferate and differentiate to form the adult abdominal tracheal system (Whitten, 1957; Samakovlis et al., 1996; Weaver and Krasnow, 2008; Pitsouli and Perrimon, 2010, 2013; Djabrayan et al., 2014). Remarkably, although SB tracheoblasts are present in all abdominal metameres of the larvae (Tr4–Tr9), only those from the Tr4 and Tr5 metameres proliferate and differentiate during metamorphosis to generate the definitive adult abdominal airways (Pitsouli and Perrimon, 2010, 2013). However, the molecular mechanisms underlying the spatially restricted proliferation of Tr4 and Tr5 SB tracheoblast remains elusive.

Upon activation, tracheoblasts in the Tr4 and Tr5 SBs start to proliferate. However, tracheoblast mitotic activity in the SBs does not occur uniformly. Instead, four cell populations with different proliferation rates can be distinguish (Pitsouli and Perrimon, 2010, 2013). The tracheoblasts located in the intermediate SB zone, called zone 2, present the higher rate of proliferation. After division, these cells move toward zone 1, the most dorsal part of the SB closest to the DT, stop proliferating and initiate one round of DNA replication by activation of the anaphase-promoting complex/cyclosome (APC/C) activator Fizzy-related (Fzr) to become 4C at the wandering stage (Pitsouli and Perrimon, 2010). Finally, tracheoblasts at zone 3 present a very low mitotic rate, while cells located at zone 4, at the most ventral tip of the SBs, do not proliferate (Pitsouli and Perrimon, 2010). Previous work has shown that the difference in the proliferation rate of the SB tracheoblasts depends on the relative abundance of the homeobox transcription factor Cut (Ct). Thus, whereas the highly proliferative zone 2 requires intermediate Ct amounts, the non-proliferative zone 1 demands the complete absence of that transcription factor (Pitsouli and Perrimon, 2013). This work also shows that the different levels of Ct result from the positive and negative regulatory activity of the Wingless and Notch signaling pathways, respectively (Pitsouli and Perrimon, 2013). Surprisingly, this particular complex expression pattern of Ct is detected in all abdominal SBs, from Tr4 to Tr9, thus suggesting that other factors must act to spatially restrict cell proliferation and differentiation to only Tr4 and Tr5 SB tracheoblasts.

To address this question, we focus on the Bnl–FGF signaling pathway, as it has been shown that the FGF receptor Breathless (Btl) is expressed in the endoreplicative cells of zone 1 as well as in the proliferative growth zone 2 of all SBs (Pitsouli and Perrimon, 2010). The expression of *btl* has been linked to the migration of the Tr4 and Tr5 tracheal progenitors into the posterior part of the abdomen later on pupal development (Chen and Krasnow, 2014). Here, we found that Bnl/Fgf signaling also exerts a dual regulatory role in the control of tracheoblast development. First, we showed that activation of Bnl/Fgf signaling in the Tr4 and Tr5 SBs is required to initiate and promote tracheoblast proliferation at zone 2. Remarkably, we show that specific tracheoblast proliferation of Tr4 and Tr5 SBs is due to the spatially restricted expression of the Fgf ligand *bnl* to these specific metameres. Importantly, we showed that Bnl/Fgf signaling also promotes endoreplication in differentiated zone 1 SB progenitor cells that express *fzr*. Finally, we demonstrate that the dual regulatory effect induced by Bnl/Fgf is transduced via the transcription factor Pointed (Pnt). Altogether, our results demonstrate that the Bnl/Fgf pathway is critical for SB tracheoblasts development, playing a dual role on promoting mitotic cell division as well as cell growth through endoreplication.

## Results

### Bnl/Fgf signaling promotes cell proliferation in SB cells

**Figure 1:**
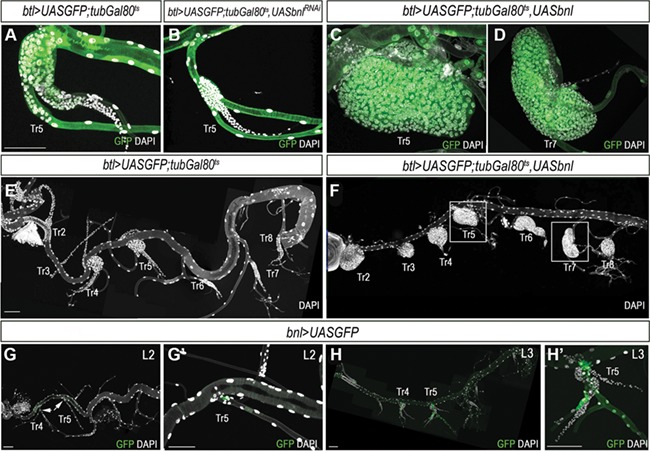
Bnl/FGF signaling activation initiates SB development. (**A**) *btl>UASGFP;tubGal80^TS^* control Tr5 SB, stained for GFP (green) and DAPI (gray). (**B**) Reduction in the number of tracheoblast cells in Tr5 SB of late L3 larvae upon depletion of FGF ligand *bnl*. (**C** and **D**) Tr5 (**C**) and Tr7 (**D**) SB cells overexpressing the FGF ligand *bnl* under the control of *btl>UASGFP;tubGal80^TS^*. (**E**) *btl>UASGFP;tubGal80^TS^* control whole tracheal system showing all SB marked with DAPI (gray). Note that only Tr4 and Tr5 SB develop. (**F**) Whole tracheal system of late L3 larvae overexpressing *bnl*. Note that overactivation of Bnl/FGF signaling induces the development of all SB. (**G**–**H′**) Expression pattern of the *bnlGal4* reporter visualized by GFP (green) at L2 (**G** and **G′**) and late L3 (**H** and **H′**). DAPI is in gray. Scale bar, 100 μm.

In order to analyze the role of Bnl/Fgf signaling in SB development, we either overactivated or inactivated the pathway in the tracheal system at L3. Depletion of the FGF ligand *bnl* in all tracheal cells using the *btlGal4* driver completely abolished proliferation of SB progenitor cells (Figure 1A and B). Consistently, overexpression of *bnl* using the same driver promoted overproliferation of SB progenitor cells and the overgrowth of the SB (Figure 1A and C). Interestingly, SB overgrowth was not restricted to Tr4 and Tr5 tracheal metameres, the two unique segments that develop during metamorphosis but to all tracheal metameres (Figure 1A–F). Similar results were obtained using a constitutive activated form of the FGF receptor Btl (Tor-Btl) (Supplementary Figure S1). Altogether, these results suggest that growth of Tr4 and Tr5 tracheal metameres might be due to Bnl being present only in these metameres. To check this possibility, we analyzed the expression of *bnl* in the larval trachea using a specific enhancer trap line that recapitulates its expression (Chen and Krasnow, 2014). As expected, *bnl* expression was restricted to the transverse connective branch of Tr4 and Tr5 during L2 and L3 stages (Figure 1G–H′).

**Figure 2:**
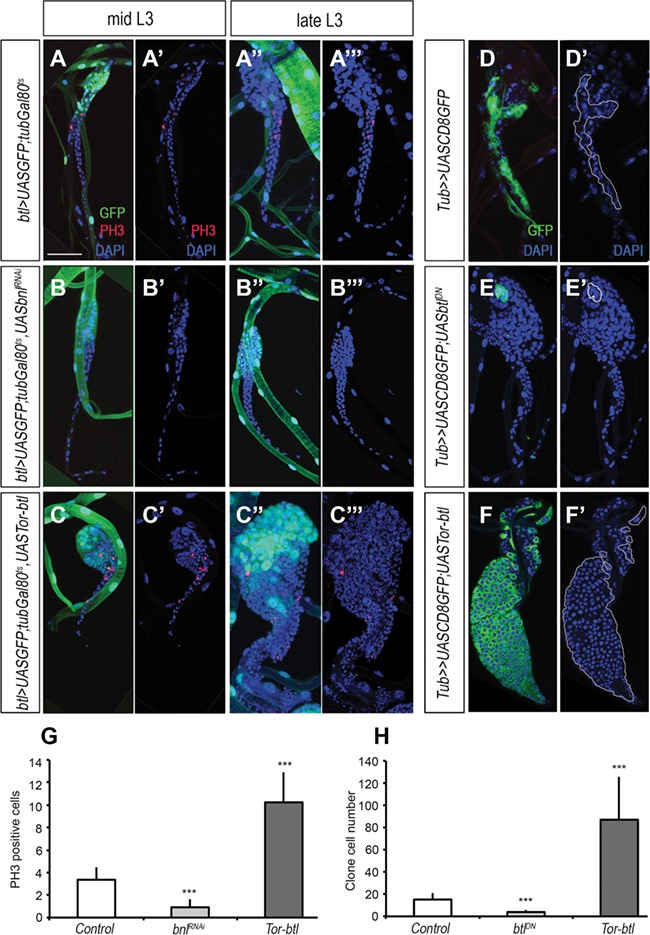
Bnl/FGF signaling induces SB adult progenitor cells proliferation. (**A**–**A′′′**) Control *btl>UASGFP;tubGal80^TS^* Tr5 SB of early and late L3 larva stained for GFP (in green), PH3 (in red), and DAPI (in blue). (**B**–**B′′′**) Tr5 SB of early and late L3 larva depleted of *bnl*. Note the lack of PH3 positive cells compare to control. (**C**–**C′′′**) Tr5 SB of early and late L3 larva overexpressing a constitutive active form of the FGF receptor *btl* (*Tor-btl*). (**D** and **D**′) SB with flip-out control clones visualized by GFP. DAPI is shown in blue. (**E** and **E′**) SB with an overexpressing clone of a dominant-negative form of the FGF receptor Btl (*UASbtl^DN^*). (**F** and **F′**) SB with an overexpressing clone *UASTor-btl*. White lines delineate clone boundaries in **D′**, **E′**, and **F′**. (**G**) Graph showing the average number of PH3-positive cells in the SBs of WT larvae and larvae ectopically expressing *UASbnl^RNAi^* or *UASTor-btl* under the control of *btl>UASGFP;tubGal80^TS^* (Student’s *t*-test, *n* > 10 SBs; ***P < 0.0001). (**H**) Graph showing the average number of either *UASGFP*, *UASbtl^DN^*, or *UASTor-btl* overexpressing clone cells (Student’s *t*-test, *n* > 10 SBs; ***P < 0.0001). Scale bar, 50 μm in **A**–**C′′′** and **D**–**F**′.

We then analyzed whether activation of Bnl/Fgf signaling in SB tracheoblasts induces cell growth or cell division. The highest rate of proliferation in the SBs takes place in zone 2 cells by mid-late L3 (Pitsouli and Perrimon, 2010). Using phospho-histone H3 (PH3), which labels mitotic cells in the G2/M transition (Shibata et al., 1990), we measured the proliferation rate of zone 2 SB cells in mid and late L3 under different conditions of Bnl/Fgf signaling activity. In the control, PH3-positive cells were detected in zone 2 in mid and late L3, which will later generate a pool of differentiated cells in zone 1 (Figure 2A–A′′′ and G). In contrast, inactivation of Bnl/Fgf signaling by depletion of *bnl* in all tracheal cells resulted in a complete absence of mitotic cells and a reduced number of SB cells by late L3 (Figure 2B–B′′′ and G). Consistently, overexpression of the constitutive activated form of *btl* receptor, *Tor-btl* in the SB produced an increase of PH3-positive cells and the consequent significant increase of differentiated SB cells by late L3 (Figure 2C–C′′′ and G). We confirmed these results by clonal analysis. Thus, *btl* dominant-negative (*btl^DN^*) overexpressing clones were significantly smaller and less abundant than control clones (Figure 2D–E′ and H), whereas clone cells overexpressing *Tor-btl* were bigger and more abundant than wild-type clones (Figure 2F, F′, and H). Altogether, these results strongly suggest that Bnl/Fgf signaling is necessary and sufficient to induce proliferation of the SB tracheoblasts.

**Figure 3:**
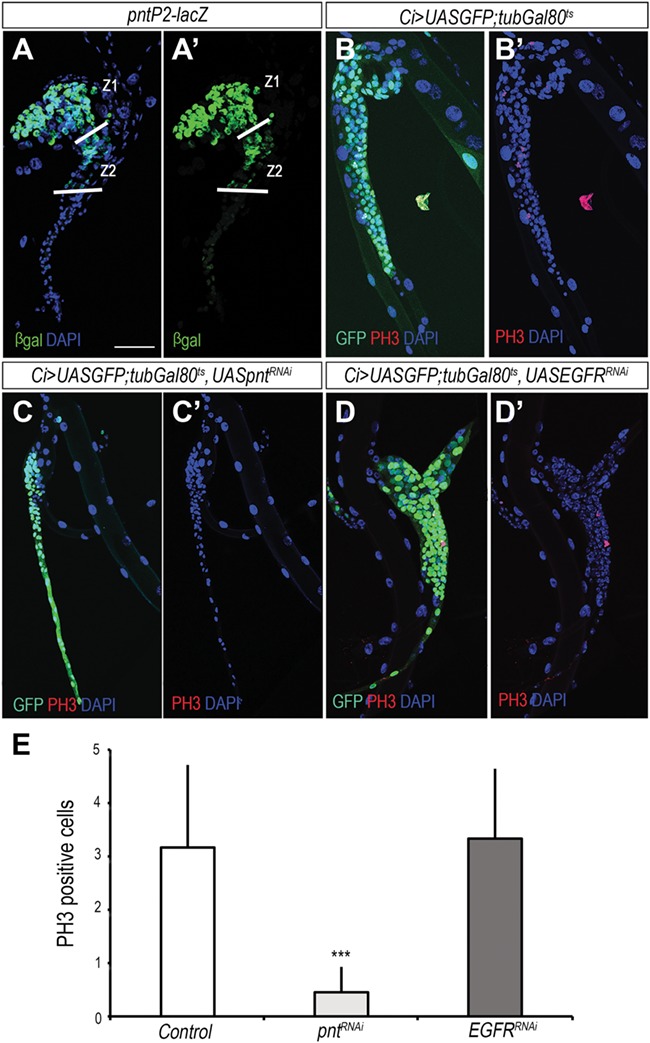
Bnl/FGF signaling acts on tracheoblast proliferation through Pnt. (**A** and **A′**) Expression of the *pntP2* reporter line in the SB visualized by anti βgal. DAPI is shown in blue. (**B** and **B′**) Control *Ci>UASGFP;tubGal80^TS^* Tr5 SB labeled with GFP in green, PH3 in red, and DAPI in blue. (**C** and **C′**) SBs depleted of *pnt* by overexpression of *pnt^RNAi^* are unable to develop. (**D** and **D**′) Overexpression of *UASEGFR^RNAi^* under control of *Ci>UASGFP;tubGal80^TS^* in the SB. (**E**) Plot depicting the average number of PH3-positive cells in the SBs of `control’ larvae and larvae ectopically expressing either *UASpnt^RNAi^* or *UASEGFR^RNAi^* under the control of *Ci>UASGFP;tubGal80^TS^* (Student’s *t*-test, *n* > 10 SBs; ***P < 0.0001). Scale bar, 50 μm in **A**–**D**.

We next investigated whether Bnl/Fgf signaling in tracheoblast proliferation requires transcriptional regulation. To address that, we analyzed the expression of *pnt*, the Ets domain transcription factor that mediates Bnl/Fgf signaling transcription activity in embryonic and larval tracheal cells (Sutherland et al., 1996; Ohshiro et al., 2002; Myat et al., 2005; Cruz et al., 2015). Using the specific enhancer trap *pnt-lacZ*, we found that *pnt* was specifically expressed where Bnl/Fgf signaling was presumably active in cells of zones 1 and 2 of the SB (Figure 3A and A′). To confirm that the expression of *pnt* is related to Bnl/Fgf signaling activation, we overexpressed *pnt^RNAi^* in SB progenitor cells under the control of *CiGal4*, a specific driver of SB cells. Interestingly, we found that depletion of *pnt* impaired SB growth by reducing cell proliferation (Figure 3B–C′ and E). Similar results were obtained when overexpressing *pnt^RNAi^* flip-out clones were generated in the SB, as low number of small clones were detected (data not shown). In addition, the overproliferation phenotype induced by either ectopic expression of *Tor-btl* or *bnl* was also suppressed by *pnt^RNAi^* overexpression (Supplementary Figure S2A–E), thus suggesting that Pnt mediates Bnl/Fgf signaling in the SB. It is important to note, however, that Pnt also transduces the activation of the epidermal growth factor (EGF) pathway, inducing mitotic division of the air sac primordium (ASP) tracheal cells through the phosphorylated isoform of Pnt, PntP2 (Cabernard and Affolter, 2005; Cruz et al., 2015). To see whether this mechanism also operates in SBs, *UAS-EGFR^RNAi^* was overexpressed under the control of the *CiGal4* driver. Interestingly, depletion of EGFR in the SB did not impair proliferation (Figure 3D, D′, and E) suggesting that, in contrast to the ASP, SB cell proliferation is only controlled by the activation of the Bnl/Fgf signaling pathway through Pnt.

### Bnl/Fgf signaling acts independently of the transcription factor *ct*

Our results above provide compelling evidence for the role of Bnl/Fgf signaling in promoting tracheoblast proliferation in the SBs. Previous studies, however, have proposed the transcription factor Ct as the main factor that coordinates cell proliferation in SBs (Pitsouli and Perrimon, 2010, 2013). It is plausible, therefore, that Bnl/Fgf signaling controls tracheoblasts proliferation by regulating *ct* expression. To analyze this possibility, we overactivated or inactivated the Bnl/Fgf signaling pathway in SB tracheoblasts and analyzed the expression of *ct* in those cells. Interestingly, Ct expression was not affected under any of these conditions (Supplementary Figure S3), suggesting that Bnl/Fgf signaling promotes proliferation in Tr4 and Tr5 SBs without affecting *ct* expression.

**Figure 4:**
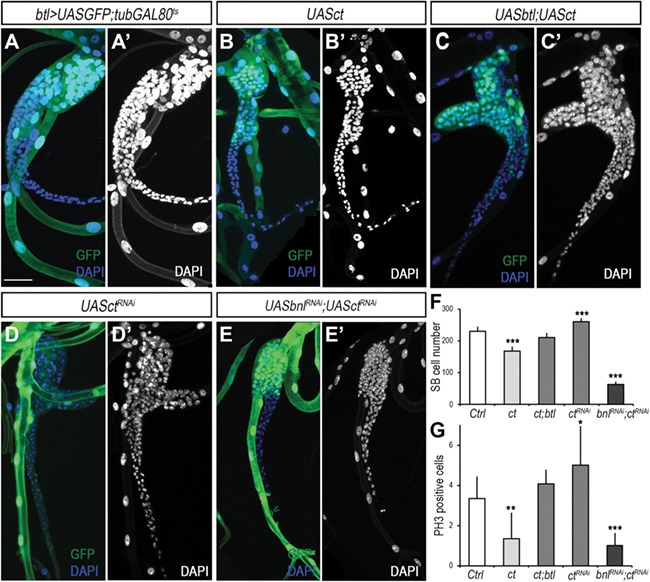
Bnl/Fgf signaling acts independently of transcription factor *ct.* (**A** and **A′**) Control *btl>UASGFP;tubGal80^TS^* Tr5 SB of late L3 larva. (**B** and **B′**) Overexpression of *ct* under control of *btl>UASGFP;tubGal80^TS^* in the SB. (**C** and **C′**) Rescue of induced *ct* depletion proliferation defect by *UASbtl*. (**D** and **D′**) SB cells depleted of *ct* by overexpression of *UASct^RNAi^*. (**E** and **E′**) Inactivation of Bnl/FGF signaling in SB cells depleted of *ct* is unable to proliferate. In all pictures GFP is green and DAPI in blue and gray. (**F** and **G**) Quantification of the total number of SB cell (**F**) and PH3-positive cells (**G**) in the anteriorly described conditions compared to control (Student’s *t*-test, *n* > 10 SBs; ***P < 0.0001). Scale bar, 50 μm in **A**–**E′**.

**Figure 5:**
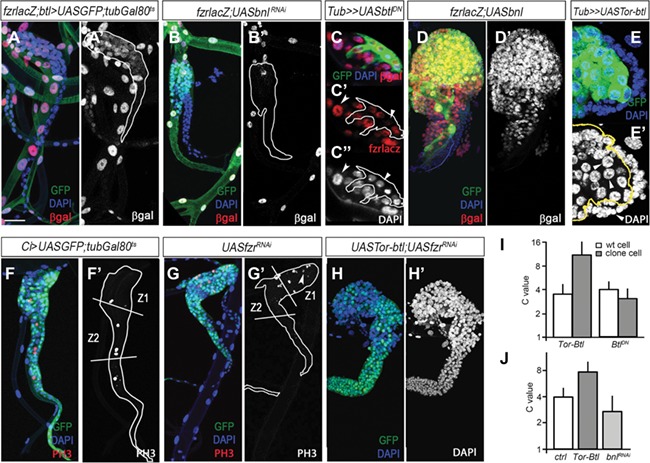
Bnl/Fgf signaling promotes endoreplication in SB differentiated cells. (**A** and **A′**) *fzr-lacZ* reporter expression in SB of late L3 larva (in red and gray) showing SB differentiated cells. DAPI is in blue. (**B** and **B′**) *fzr-lacZ* expression in SB of late L3 tracheal system depleted of *bnl*. White lines delineate SB shape in **A′** and **B′**. (**C**–**C′′**) *fzr-lacZ* expression in clone cells overexpressing *UASbtl^DN^* visualized by GFP. βgal is shown in red and DAPI in gray. Note the reduction of *fzr* expression of clone cells and nuclear size compared to adjacent control cells (arrowheads). White lines delineate clone boundaries in **C′** and **C′′**. (**D** and **D′**) *fzr-lacZ* expression in SB cells overexpressing the FGF ligand *bnl*. (**E** and **E′**) Flip-out clone overexpressing *UASTor-btl* marked by the expression of GFP. Increased of the nucleus size, visualized by DAPI (in blue and gray) is observed in clone cells compared to the surrounding control cells (arrowheads). Clone boundaries are outlined with a yellow line. (**F** and **F′**) Control *Ci>UASGFP;tubGal80^TS^* Tr5 SB of late L3 larva marked with GFP (in green) and stained for mitotic marker PH3 (red and gray). (**G** and **G′**) SB cells depleted of *fzr*. Note the increase of PH3-positive cells in the SB differentiated cells of zone 1 (arrowhead). SB morphology is outlined with a white line. (**H** and **H′**) Overexpression of *UASTor-btl* in the Tr5 SB depleted of *fzr*. (**I**) Quantification of the C value of clone cells in zone 1 of SB overexpressing either *UASTor-btl* or *UASbtl^DN^* compared to surrounding control cells. (**J**) Graph showing the C value of zone 1 SB cells overexpressing either *UASTor-btl* or *UASbnl^RNAi^*. Scale bar, 50 μm in **A**–**B′′**, **D**–**D′′**, and **F**–**H′**.

Then, we investigate whether *ct* expression is necessary for Bnl/Fgf signaling activity to maintain proliferation of the SB cells. In fact, it has been shown that Ct restricts the expression of the *btl* to cells in zones 1 and 2 of the SB (Pitsouli and Perrimon, 2010, 2013). According to this regulation, a reduction of *ct* expression induces an increase of Btl levels in zone 2, promoting cell proliferation in this area. Conversely, overexpression of *ct* would reduce cell division in zones 1 and 2 by repressing *btl* expression. To verify this hypothesis, we either overexpressed or depleted Ct in the SB by using the *btlGal4*; *tubGAL80^ts^* driver, and rearing the animals at 25°C to avoid cell death as Ct acts as a cell survival factor in the SB (Pitsouli and Perrimon, 2013). As predicted, overexpression of *ct* in tracheoblasts reduced SB cell total number and PH3-positive cells due to a repression of *btl* expression (Figure 4A–B′, F, and G; Supplementary Figure S4A–C). Interestingly, we rescued the cell number defect induced by the ectopic expression of *ct* by co-expressing the FGF receptor *btl* (Figure 4C, C′, F, and G). Similar effect was obtained when flip-out clones overexpressing *UASct* and *UASbtl* were generated in the SB (Supplementary Figure S5A–C). In addition, co-overexpression of *UASct* and the constitutive activated form of the receptor Btl, *UASTor*-*btl* amplifies the mitotic effect, increasing dramatically the cell number of the clones (Supplementary Figure S5D). In contrast, partial depletion of *ct* to allow tracheoblast survival increased PH3-positive cells and consequently SB cell number (Figure 4D–D′, F, and G). This positive effect of *ct* knockdown in proliferation of the SB cells is due to a higher expression of *btl* in zone 2 (Supplementary Figure S4D), and consequently inactivation of the pathway in *ct* depleted tracheal cells by co-expressing *UASbnl^RNAi^* reduced dramatically the number of SB cells (Figure 4E–G). Conversely, overactivation of the Bnl/Fgf pathway in SB clone cells partially depleted of *ct* resulted in a higher overproliferation of these cells (Supplementary Figure S5E and F), confirming that the effect of Ct on proliferation depends on the regulation of *btl* expression. Altogether, we conclude that Ct expression in SB cells is only required to restrict the population of SB cells that express *btl* receptor but not to initiate tracheoblast proliferation.

### Bnl/Fgf signaling pathway promotes endoreplication in SB differentiated cells

As described above, activation of Bnl/Fgf signaling pathway in tracheoblasts of zone 2 induces cell proliferation. However, upon entering into zone 1, where Btl is present at high levels, these cells stop proliferating and initiate one round of endoreplication (Pitsouli and Perrimon, 2010). This transition depends on the repression of Ct by the Notch signaling, which allows the specific expression of the endocycle marker Fzr in SB cells of the zone 1 (Figure 5A; Pitsouli and Perrimon, 2013). Fzr is a Cdh1-like positive regulatory subunit of the APC/C that induces the degradation of the mitotic cyclins in G1, thus promoting endoreplication (Sigrist and Lehner, 1997; Jacobs et al., 2002). As Bnl/Fgf signaling pathway is active in zone 1 SB cells, it is possible that this pathway could also promote endoreplication. To study the potential role of Bnl/Fgf signaling pathway in the endoreplication of SB cells, we first analyzed the expression of *fzr* in cells of zone 1 with overactivation and inactivation of the pathway. Inactivation of the pathway by depleting *bnl* in all tracheal cells abolished the expression of *fzr-lacZ* (Figure 5A–B′). However, we cannot discard that the impaired expression of *fzr* was due to the fact that inactivation of Bnl/Fgf signaling in SB cells prevents the development of the SB. To avoid this problem, we generated flip-out clones overexpressing *btl^DN^* and found that clone cells with inactivated Bnl/Fgf signaling presented reduced expression of *fzr-lacz* and smaller nucleus with less genomic DNA as measured by their chromatin value (C value), when compared to their control counterpart cells (Figure 5C–C′′ and I). In contrast, overactivation of Bnl/Fgf signaling by overexpression of *bnl* in all tracheal cells increased the expression level of *fzr* in SB cells, which underwent one extra round of endocycle resulting in an increase in the C value from 4C to 8C (Figure 5D, D′, and J). A higher rate of endoreplication was also observed when flip-out clones overexpressing *UASTor-btl* were generated in the SB cells, as clone cells presented bigger nucleus and increased DNA content (8C) than the surrounding control cells (4C) (Figure 5E, E′, and I). These results suggest that the Bnl/Fgf signaling pathway promotes either proliferation or endoreplication of SB tracheoblasts depending on the absence or presence of Fzr, respectively. If this were the case, depletion of *fzr* would result in an increase of mitotic activity in zone 1 cells. Confirming this possibility, depletion of *fzr* in the SB induced a switch from endoreplication to cell proliferation in zone 1 cells and the consequent reduction of cell size when *fzr^RNAi^* was overexpressed under the control of *CiGal4* (Figure 5F–G′). Consistently, overactivation of Bnl/Fgf signaling activity in the absence of Fzr dramatically increased the number of non-endoreplicative cells in zone 1 (Figure 5H and H′). Altogether, our results demonstrate that the Bnl/Fgf signaling pathway increases cell proliferation in Fzr-absent SB cells of zone 2, whereas it promotes endoreplication in SB cells of zone 1 that express *fzr*.

## Discussion

Our analysis of Bnl/Fgf signaling on SB morphogenesis shows that (i) the activation of the pathway in the Tr4 and Tr5 SBs is required and sufficient to initiate the normal development of these SBs, (ii) the action of Bnl/Fgf signaling promotes either proliferation or endoreplication of the SB cells depending on the expression of *fzr*, and (iii) Ct regulates the SB cell behavior mode by targeting *fzr* at the transcriptional level, but it is dispensable for Bnl/Fgf signaling activity. Our results illustrate how the activation of the Bnl/Fgf pathway along the SBs induces two different processes depending on the genetic context of the cell, proliferation or endoreplication. Our data also demonstrate that the activation of Bnl/Fgf signaling, rather than the levels of Ct, controls the initiation of SB development and cell proliferation (Supplementary Figure S6).

Bnl/Fgf signaling in *Drosophila* has been described as the main pathway that guides and differentiates tracheal cells during embryogenesis to form the larval trachea and also during metamorphosis to remodel and form the definitive adult trachea (Samakovlis et al., 1996; Sato and Kornberg, 2002; Ghabrial et al., 2003; Cabernard and Affolter, 2005; Guha and Kornberg, 2005; Guha et al., 2008; Sato et al., 2008; Weaver and Krasnow, 2008; Pitsouli and Perrimon, 2010; Cruz et al., 2015). In addition to these well-established roles, our results describe for the first time a role of Bnl/Fgf signaling in SB development by promoting cell proliferation and endoreplication. To date, the transcription factor Ct was considered the factor that promotes either cell proliferation or differentiation in SBs depending on its relative abundance (Pitsouli and Perrimon, 2013). Our work, however, provides several lines of evidence demonstrating that it is Bnl/Fgf signaling activity, rather than Ct, that is responsible for promoting SB cell proliferation and endoreplication: (i) absence of Bnl/Fgf signaling activity abolished SB development even in the presence of *ct* expression; (ii) ectopic activation of the Bnl/Fgf pathway induces SB growth by the dramatic increase of tracheoblast proliferation; (iii) whereas *ct* is similarly expressed in the SB of every tracheal metamere, *bnl* is specifically expressed only in the two metameres that will grow and develop, namely Tr4 and Tr5 metameres (Figure 1G–H′); and (iv) overexpression of *bnl* in all tracheal cells initiates SB development in all metameres (Figure 1F). In this regard, it is worth noting that the factor that induces and restricts *bnl* expression to the Tr4 and Tr5 metameres is still unknown. The regulation of *bnl* expression is very complex as its expression in the ectoderm and tracheal cells is very dynamic during development (Sutherland et al., 1996; Sato and Kornberg, 2002; Chen and Krasnow, 2014). Nevertheless, it is likely that Hox genes control the expression of *bnl* in the larva tracheal system. In this sense, Tr4 and Tr5 are specified by the expression of low levels of Abdominal A and Ultrabithorax (Guha and Kornberg, 2005; Sato et al., 2008; Djabrayan et al., 2014), a hox code that may allow the presence of Bnl. Further experiments are needed to check this hypothesis.

Although Bnl/Fgf signaling had been shown to promote proliferation of the ASP tracheoblasts (Sato and Kornberg, 2002), recent works have proved that this effect is indirect as it is mediated by the activation of the EGF signaling pathway in this tissue (Cabernard and Affolter, 2005; Cruz et al., 2015). In contrast, our data show that in the SB, Bnl/Fgf signaling promotes proliferation directly via the transcription factor *pnt* and independently of the EGF pathway (Figure 3; Supplementary Figure S2). Therefore, our data show a direct role of Bnl/Fgf signaling in proliferation in *Drosophila*, in a similar way that occurs during the mammary gland development, where FGF signaling stimulates cell proliferation to generate cells both at the branching epithelium tips and in the subtending duct (Lu et al., 2008; Parsa et al., 2008).

Contrary to previous reports, we also show that the main role of Ct in the SB is to determine the cell mode of the tracheoblast by regulating the expression of *fzr*. In this regard, our data indicate that Bnl/Fgf signaling induces cell proliferation or endoreplication depending on the presence or absence of Ct, respectively. Thus, Ct acts in the SB as in the ovary follicular epithelium where it also regulates the switch from mitotic cycles to endoreplication (Sun and Deng, 2005). The requirement for Ct in maintaining the mitotic cell cycle in *Drosophila* tracheoblast echoes its role in mammalian systems. The data in mammals suggest that CDP/Cut expression or activity might be restricted to proliferating cells (Sansregret and Nepveu, 2008). Interestingly, the expression of the mouse CDP/Cut protein Cux-1 in the kidney was found to be inversely related to the degree of cellular differentiation (Vanden Heuvel et al., 1996). In addition, it has been shown that depletion of Cux-1 resulted in a significant increase in binucleate hepatocytes (Wang et al., 2017).

Interestingly, our data indicate that Bnl/Fgf pathway not only initiates SB development and promotes the proliferation of tracheoblasts but also promotes endoreplication in these cells. In zone 1 of the SB, the activation of Notch signaling represses *ct* expression, thus allowing the initiation of endoreplication through the upregulation of the APC activator Fzr/Cdh1 (Shcherbata et al., 2004). Once activated, successive endocycle rounds are regulated by an intrinsic oscillator that consist of alternate APC activator and the levels of Cyc E (Zielke et al., 2013). Depending on the number of the times that the oscillator is on cells reach 4C, 8C, 16C, 32C, etc. The activity of the Bnl/Fgf pathway seems to regulate the oscillator in SB differentiated cells, as inactivation of the pathway reduces DNA content, whereas its overactivation increases the number of endocycles. Interestingly, this dual effect depending on the cell context is reminiscent of the EGF signaling in the adult gut. After gut epithelial damage, EGF signaling drives proliferation of intestinal stem cells (ISCs), as well as endocycling in differentiated enterocytes (Xiang et al., 2017). As EGF and Bnl/Fgf pathways share many downstream components, including the transcription factor *pnt*, it is conceivable that the mechanism to promote endoreplication might be similar. Another example is found in the oncogene *Dmyc*, which stimulates cell proliferation of ISCs in the *Drosophila* adult midgut (Ren et al., 2013) as well as endoreplication of fat body cells (Pierce et al., 2004). The hippo pathway has also been involved in promoting cell proliferation or endoreplication in larval tracheal cells depending on the expression of *fzr* (Djabrayan et al., 2014). However, the role of Bnl/Fgf pathway in SB development contrasts with the effect of FGF4 in mammals. In this case, FGF is only required to maintain the proliferation of trophoblast stem cells, as its inactivation drives to the formation of trophoblast giant cells that growth by endoreplication (Ullah et al., 2008).

Our observations of the *Drosophila* tracheal system reveal that one signaling pathway can be used in a specific developmental process to induce both cell proliferation and endocycling and that this capacity may be more common than has been generally appreciated not only in development but also in cancer. In certain contexts, cancerous cells use endoreplication as a path to drug resistance (Shen et al., 2008; Sakaue-Sawano et al., 2011). Interestingly, different evidences point to the upregulation of the FGF/FGFR signaling as a mechanism of chemo and radio-resistance in cancer therapy (Presta et al., 2017). Future studies should prove a possible link between FGF/FGFR system and endoreplication in tumors and promise insight into how to treat therapy resistance cancers.

## Materials and methods

### Fly stocks

Details for all strain genotypes can be obtained from FlyBase (http://flybase.org) or in references listed here. Conditional activation of either RNAi or gene expression was achieved using the *Gal4/Gal80^ts^* System (McGuire et al., 2004). To overexpress UAS transgenes either in all tracheal cells or in SB cells, *btlGal4UASGFP*;*tubGal80^ts^* or *CiGal4;tubGal80^ts^* was used, respectively. Crosses were kept at 18°C until late in L2 when larvae were shifted to 29°C for 48 h and dissected. The following stock flies where obtained from the Bloomington Stock Center: *btlGal4* (#8807), *tubGal80^ts^* (#7016), *UASpnt^RNAi^* HMS01452 (#35038), *UASct^RNAi^* JF03304 (#29625), *UASEGFR*^*RNA*i^ JF01368 (#25781), *fzr-lacZ^G0326^* (#12241), and *pnt^1277^(pntP2-lacZ, #837)*. *UAS-fzr^RNAi^ (*#2550 and #25553), *UASbnl^RNAi^* (#101377 and #5730), and *UASpnt^RNAi^* (#105390) were from Vienna Drosophila Resource Center (VDRC) and *CiGal4* (Croker et al., 2006), *UASbtl* (Myat et al., 2005), *UASbtl^DN^* (Reichman-Fried and Shilo, 1995), *bnlGal4* (Hayashi et al., 2002), *UASTor-btl* (Vincent et al., 1998), *UASbnl* (Sutherland et al., 1996), *btl-mRFPmoe* (Ribeiro et al., 2004), and *UASct* (Ludlow et al., 1996) were given.

### Flip-out clones

Females of the genotype: *hsflp^70^;UASCD8GFP;tub>y^+^>Gal4* where crossed with the following UAS transgenic males: *UASTor-btl*, *UASbtl^DN^*, *UASbtl*, and *UASct* were kept at 25°C until they reached early third-instar stages. After a 30 min heat shock at 37°C, the larvae were transferred back to 25°C for 14–16 h and dissected.

### Immunohistochemistry

Larval trachea was dissected at third larval instar and fixed in 4% formaldehyde for 20 min. Samples were incubated overnight at 4°C with primary antibodies and for 2 h at room temperature with secondary antibodies and then were mounted in Vectashield with DAPI (Vector Laboratories). The following primary antibodies were used: anti-Ct (2B10, 1:100) and anti-β-galactosidase (401.a, 1:200) from Developmental Studies Hybridoma Bank and anti-PH3 (1:500) from Cell Signaling Technology. Fluorescent-conjugated secondary antibodies were obtained from Molecular Probes. Images were obtained with SP5 confocal microscope and processed with either Fiji or Photoshop CS4 (Adobe).

### DNA quantification

For DNA quantification, DNA staining intensity in the SB cells was obtained from z-stacked images of DAPI stained tracheal system. The images were processed and analyzed using standard ImageJ (FIJI) measurement tools. DNA staining intensity of SB cells of zone 1 was normalized using average DNA staining intensity in the SB zone 4 cells: DNA staining intensity in the SB cells of zone 1/DNA staining intensity in SB cells of zone 4. The C value of the control SB cells of zone 1 cells at late L3 was set to 4C (Welch, 1957).

## Supplementary Material

JMCB-2018-0500-R2_Supplementary_Figures_mjz055
