# Supplementary material for: Dual role of FGF in proliferation and endoreplication of Drosophila tracheal adult progenitor cells
Source: J Mol Cell Biol. 2019 Jun 25;12(1):32–41. doi: 10.1093/jmcb/mjz055 (PMC7050688; doi:10.1093/jmcb/mjz055)
Supplement: JMCB-2018-0500-R2_Supplementary_Figures_mjz055 [file jmcb-2018-0500-r2_supplementary_figures_mjz055.pdf]

Supplementary Figures

Dual role of FGF in proliferation and endoreplication of Drosophila tracheal adult progenitor cells

Cristina de Miguel*, Josefa Cruz*, David Martín and Xavier Franch-Marro†

Institute of Evolutionary Biology (IBE, CSIC-Universitat Pompeu Fabra), Passeig Marítim de la Barceloneta 37, 08003 Barcelona, Catalonia, Spain.

† Author for correspondence: xavier.franch@ibe.upf-csic.es
*These authors contributed equally to this work.

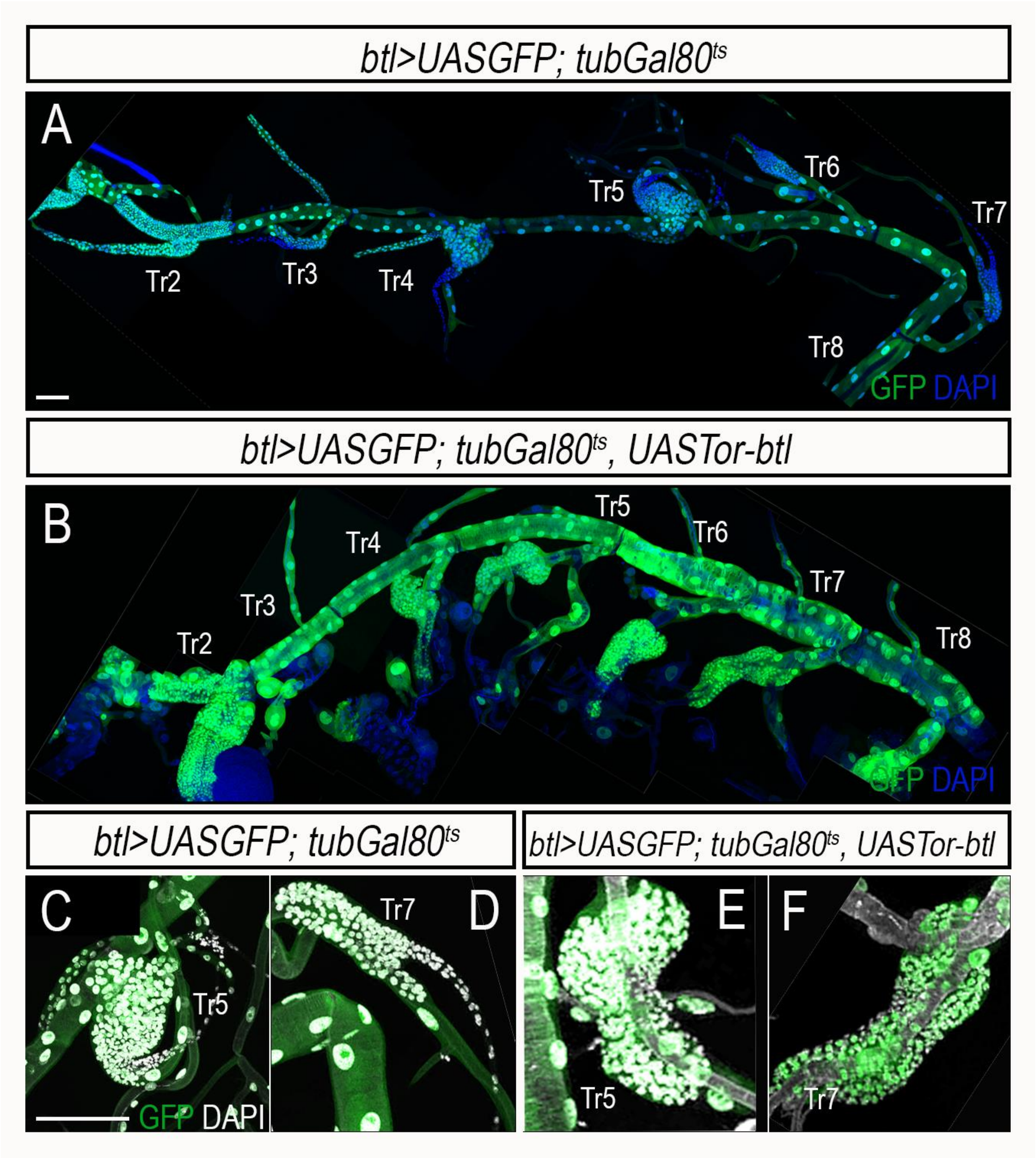

Supplementary Figure 1. Effect on SB development upon Bnl/FGF signaling overactivation. (A) Control *btl>UASGFP; tubGal80*^{TS} whole tracheal system showing all SB marked with GFP (green) and DAPI (blue). Note that only Tr4 and Tr5 SB develop. (B) Whole tracheal system of late L3 larvae overexpressing *UASTor-btl*. Note that over activation of Bnl/FGF signaling induces proliferation of all SBs. (C) Tr5 and (D) Tr7 SB control cells. (E) Tr5 and (F) Tr7 SB cells overexpressing *UASTor-btl* under the control of *btl>UASGFP; tubGal80*^{TS}. Scale bars represent 100 μm.

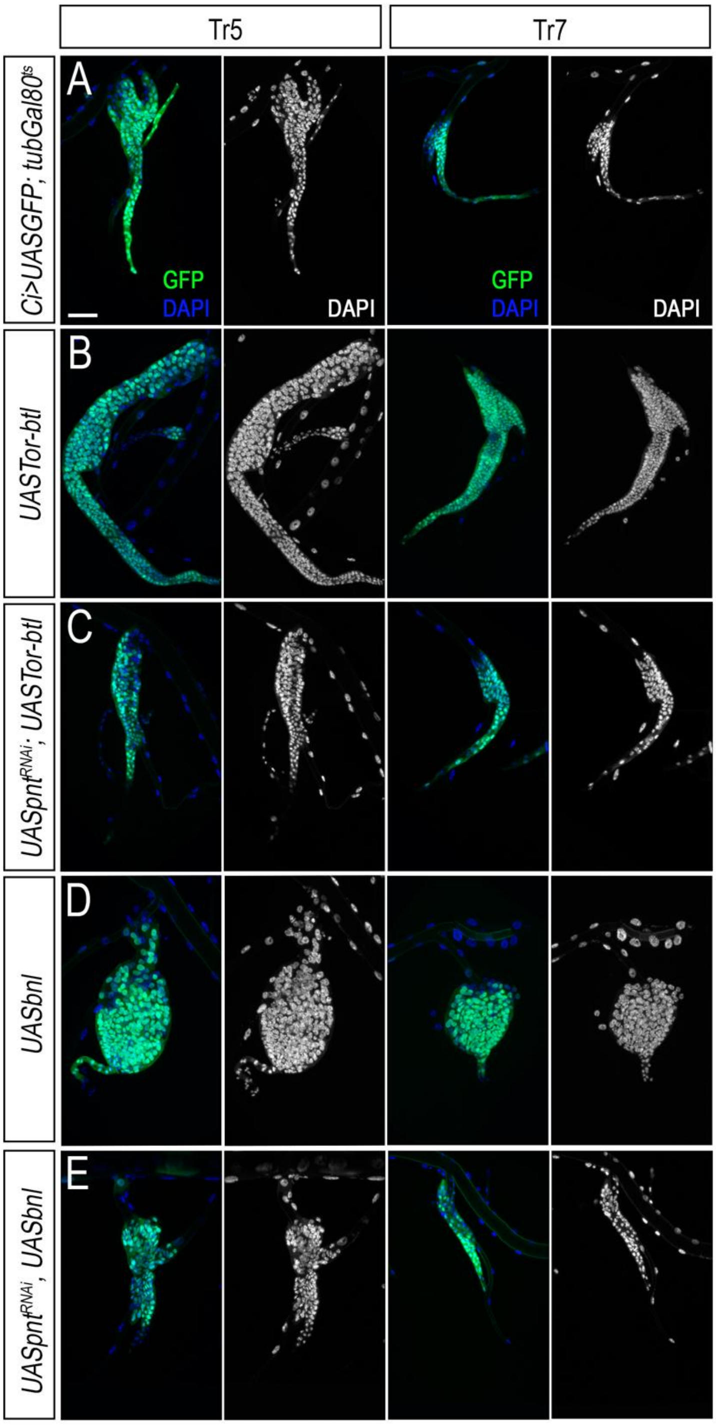

Supplementary Figure 2. Bnl/FGF signaling depends on Pnt transduction in SB cells. (A-E) Tr5 and Tr7 SBs marked with GFP (green) and DAPI (blue and grey) overexpressing under control of *CiGal4; tubGal80*^{TS} (A) *UASGFP* (*Control*), (B) *UASTor-btl*, (C) *UASTor-btl and UASpnt*^{RNAi}, (D) *UASbnl* and (E) *UASbnl* and *UASpnt*^{RNAi}. Note that depletion of *pnt* reverts the proliferative effect of the overactivation of Bnl/FGF signaling. Scale bars represent 50 μm.

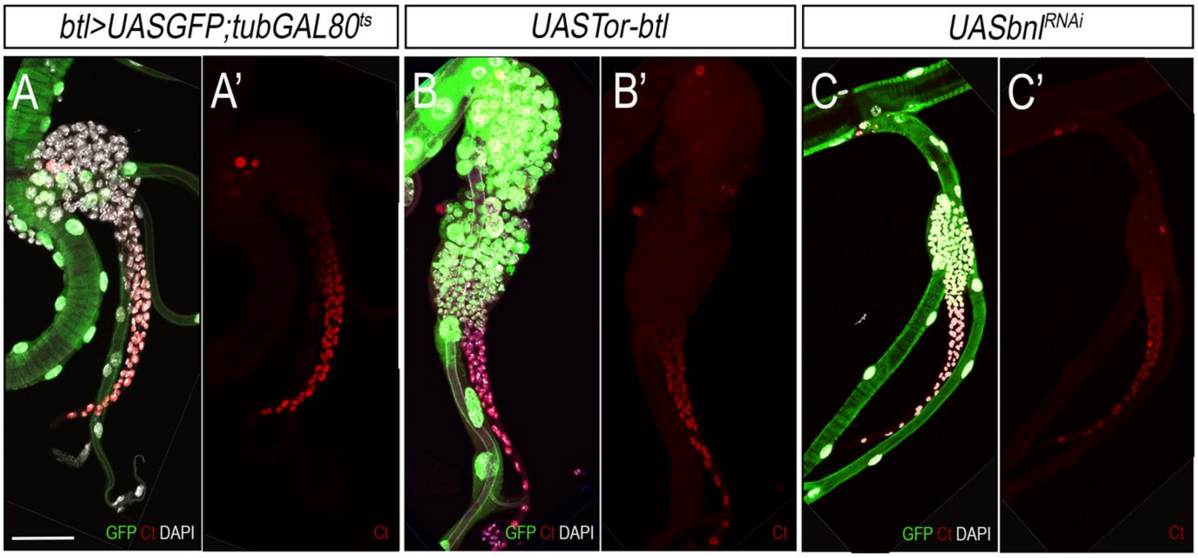

Supplementary Figure 3. Bnl/FGF signaling effect on *ct* expression. In all pictures GFP is shown in green, Ct in red and DAPI in grey. (A-A') *btl>UASGFP; tubGal80^{TS}* control SB. (B-C') SB cells overexpressing either (B-B') *UASTor-btl* or (C-C') *UASbnl^{RNAi}*. Scale bars represent 50 µm.

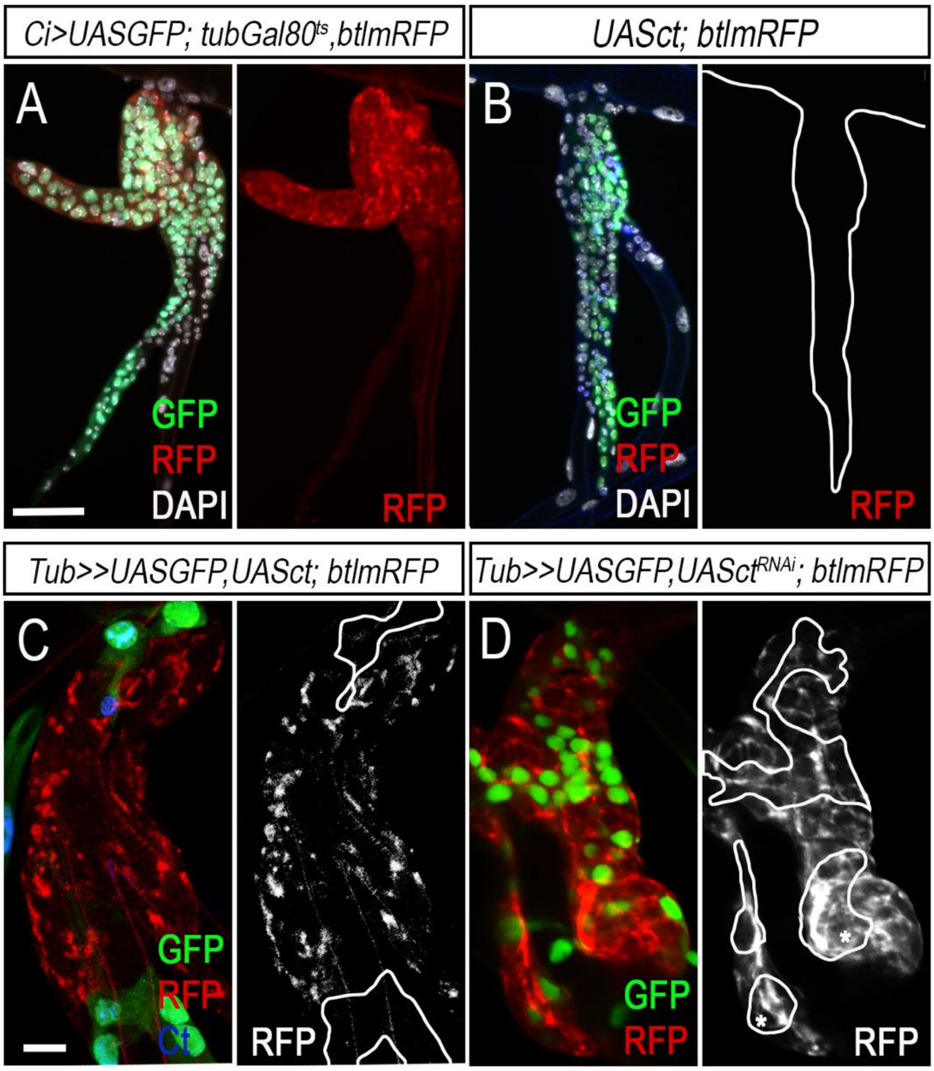

Supplementary Figure 4. Ct regulates the expression of the receptor *btl*. (A) Control SB *Ci>UASGFP; tubGal80^{TS}* revealed by GFP and DAPI (in green and grey respectively) showing the expression of *btl-mRFP* reporter in zone 1 and 2 (in red). (B) Overexpression of *ct* under the control of *Ci>UASGFP; tubGal80^{TS}* represses the expression of *btl-mRFP*. White line delineates SB shape. (C) *ct* overexpressing clones in zone 1 and 2 of SB (labelled by GFP in green and Ct in blue) showing the loss of btl-mRFP reporter expression (in red). In grey *btl-mRFP* reporter only. (D) SB clone cells overexpressing *UASct^{RNAi}* (marked by GFP) in the background of the *btl-mRFP* reporter (red). Grey shows btl-mRFP. Note that an increase in *btl-mRFP* expression is only detected in the clones located in zone 2 (asterisks), where *ct* is normally expressed. White lines delineate clone boundaries in (C) and (D). Scale bars represent 50 µm in A-B and 10 µm in C-D.

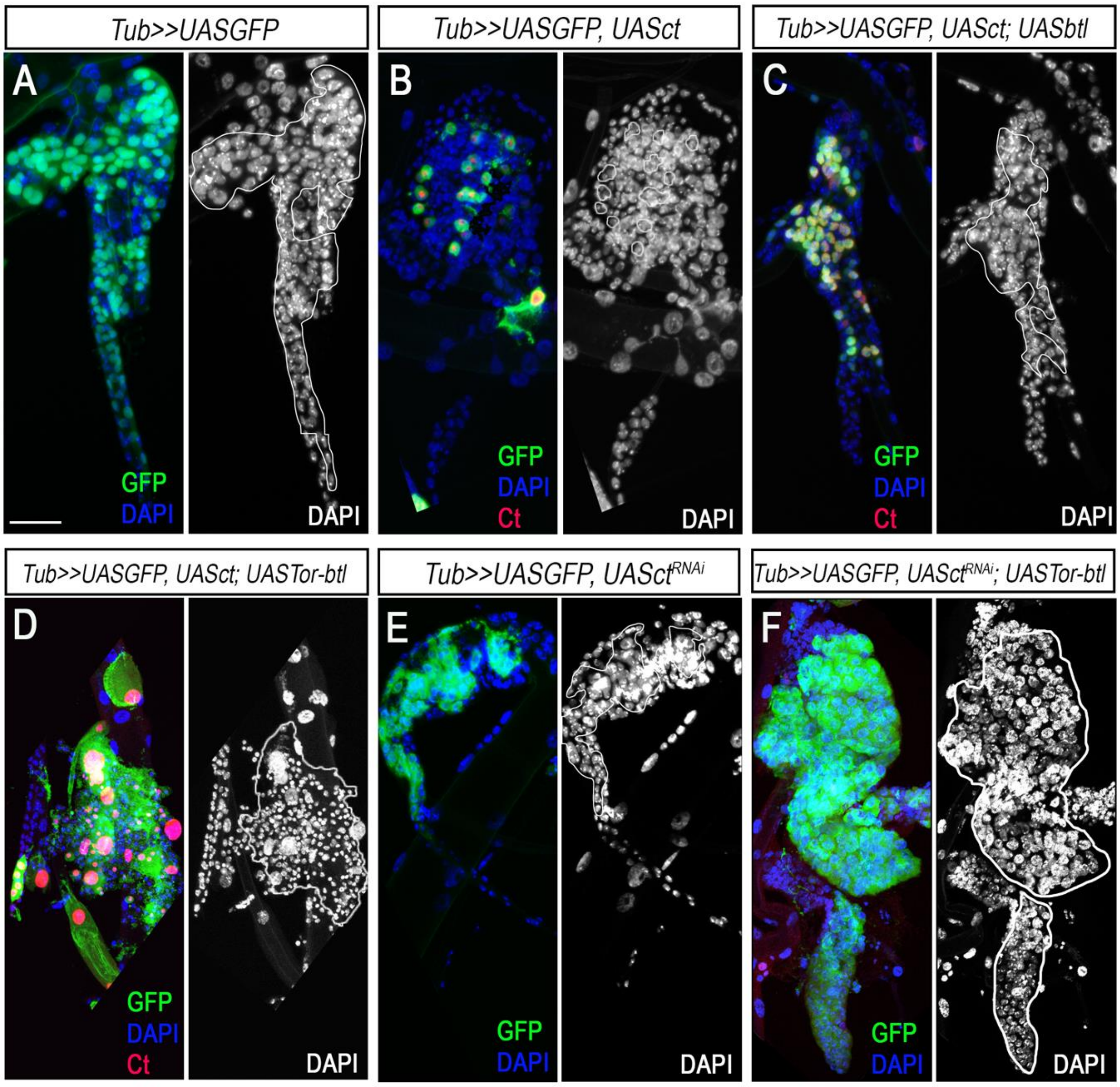

Supplementary Figure 5. Effect of Ct in Bnl/FGF signaling activation. (A) SB with flip-out control clones visualized by GFP (in green) and DAPI (in blue and grey). (B) Flip-out clones overexpressing *UASct* marked with GFP and stained for Ct (red). Nucleus are marked with DAPI (blue and grey). (C, D) Flip-out clones co-overexpressing either (C) *UASbtl and UASct or (D) UAS-Tor-btl* and *UASct*. Note that in both cases the size of the clones increased. (E) Flip-out clones marked with GFP and DAPI (blue and grey) overexpressing either only *UASct*^{RNAi} or (D) *UASTor-Btl* and *UASct*^{RNAi}. White lines delineate clone boundaries. Scale bars represent 50 μm.

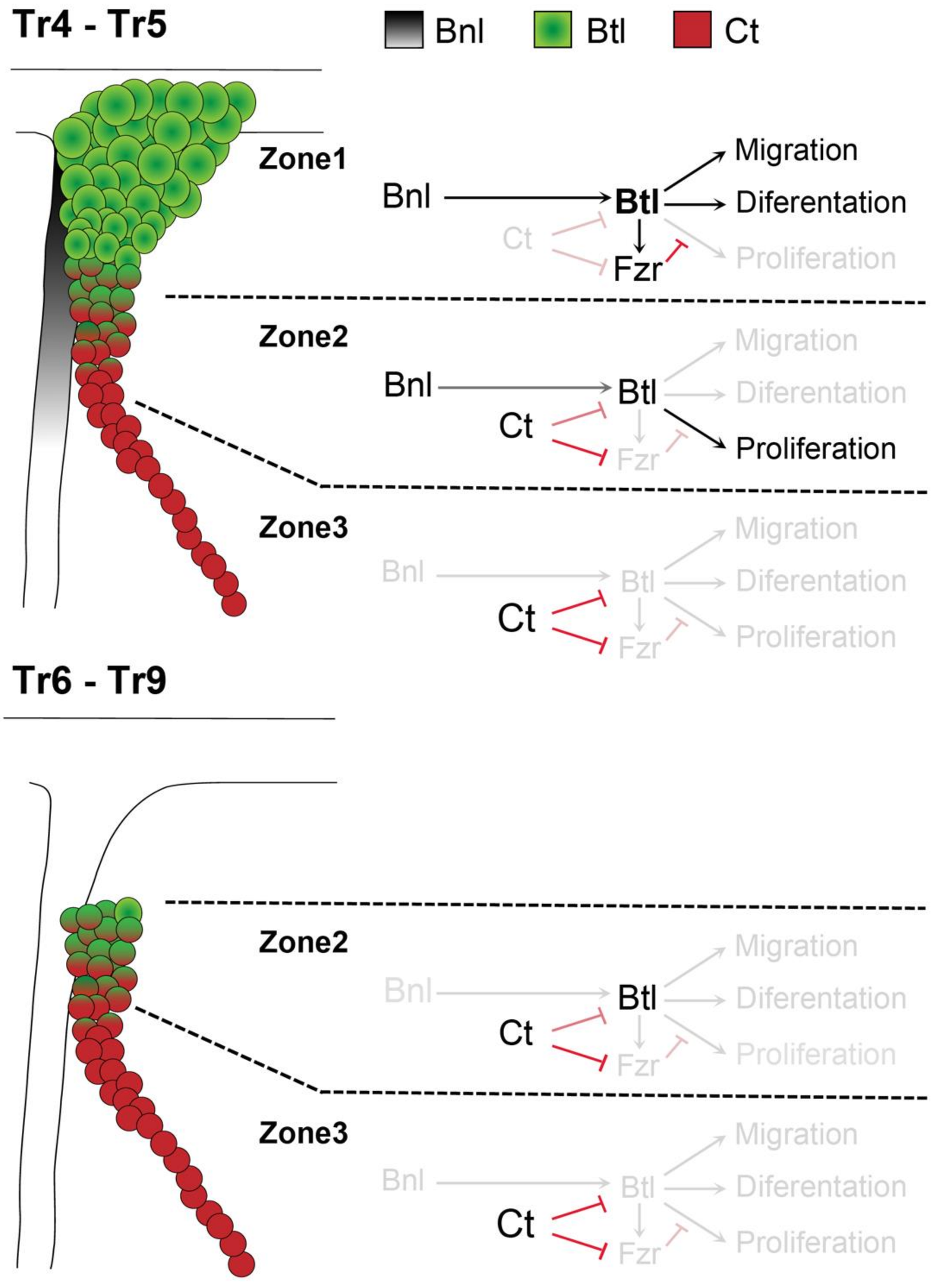

Supplementary Figure 6. Model depicting the spatial activation of Bnl/FGF signaling in adult progenitor cells. Bnl/FGF signaling is activated by the ligand Bnl specifically in Tr4 and Tr5 tracheal metamers. The different outcome of the activation of Bnl/FGF pathway, depending on the presence or absence of Ct allowing proliferation and endoreplication respectively, is also shown. In metameres Tr6 to Tr9 bnl is not present and therefore, Bnl/FGF signaling is not activated despite the presence of Ct. As a consequence, adult progenitor cells do not proliferate.
